# Tat-Interacting Protein 30 (TIP30) Expression Serves as a New Biomarker for Tumor Prognosis: A Systematic Review and Meta-Analysis

**DOI:** 10.1371/journal.pone.0168408

**Published:** 2016-12-30

**Authors:** Tao Xu, Zhichao Jin, Yuan Yuan, Honggang Zheng, Conghuang Li, Wei Hou, Qiujun Guo, Baojin Hua

**Affiliations:** 1 Department of Oncology, Guang’anmen Hospital, China Academy of Chinese Medicine Sciences, Xicheng District, Beijing, China; 2 Department of Oncology, Xiyuan Hospital, China Academy of Chinese Medicine Sciences, Haidian District, Beijing, China; 3 Beijing University of Chinese Medicine, Chaoyang District, Beijing, China; University of South Alabama Mitchell Cancer Institute, UNITED STATES

## Abstract

**Background:**

Tat-interacting protein 30 (TIP30) is a tumor suppressor protein that has been found to be expressed in a wide variety of tumor tissues. TIP30 is involved in the control of cell apoptosis, growth, metastasis, angiogenesis, DNA repair, and tumor cell metabolism. The methylation of the TIP30 promoter is also associated with tumor prognosis. To evaluate this topic further, we conducted a systematic meta-analysis to explore the clinicopathological and prognostic significance of TIP30 for tumor patients.

**Methods:**

We searched PubMed and EMBASE for eligible studies. We manually searched for printed journals and relevant textbooks. Subgroup analyses were performed based on the region, manuscript quality, methods of vasculogenic mimicry identification, pathology, and number of patients.

**Results:**

Fourteen studies with 1705 patients were included in this meta-analysis. A significant association was observed between high expression of TIP30 in patients with cancer with a good overall survival (hazard ratio = 0.53, 95% confidence interval: 0.41–0.69), and good recurrence-free survival or disease free survival (hazard ratio = 0.49, 95% confidence interval: 0.37–0.66). Lack of expression of TIP30 had an association with lymph node metastasis (odds ratio = 3.90, 95% confidence interval: 2.21–6.89) and high tumor node metastasis clinical stage (odds ratio = 2.10, 95% confidence interval: 1.68–2.62). The methylation of the TIP30 promoter did not significantly influence the overall survival (hazard ratio = 0.99, 95% confidence interval: 0.88–1.13) or disease free survival (hazard ratio = 0.62, 95% confidence interval: 0.19–2.02).

**Conclusions:**

TIP30 expression is associated with a good prognosis in patients with tumors. Clinical studies with large samples are needed worldwide and standardized protocols should be adopted in the future to achieve a better understanding of the relationship between tumor prognosis and TIP30.

## Introduction

Malignant neoplasms have a high fatality rate worldwide. Although treatment approaches such as surgical operations, chemotherapy, radiotherapy, and targeted therapy have achieved a certain therapeutic effect, there are no satisfactory treatment methods for recurrence or metastasis [1–5]. Researchers have been identifying prognostic biomarkers in tumor patients. Proteins such as vascular endothelial growth factor A (VEGF-A) [6], extracellular signal-regulated kinase (ERK) [7], matrix metalloproteinases (MMPs) [8], and pro-apoptotic protein p53 [9] were found to be associated with the overall survival time of patients and the malignant behaviors of some tumors. However, it is still necessary to explore established markers possessing predicative values for the survival of cancer patients.

Tat-interacting protein 30 (TIP30), also called CC3 or HTATIP2, is a metastasis suppressive protein, which was first found in patients with small cell lung carcinoma (SCLC) [10]. TIP30 is involved in the control of cell apoptosis, growth, metastasis, angiogenesis, DNA repair, and tumor cell metabolism [11]. TIP30 has been found to be expressed in a wide variety of tumor tissues, including esophageal carcinoma [12], laryngeal carcinoma [13], glioma [14], pancreatic ductal adenocarcinoma [15], breast cancer [16], gastric cancer [17], gallbladder adenocarcinoma [18], lung cancer [19], and hepatocellular carcinoma [20]. The methylation of the TIP30 promoter may also be associated with tumor prognosis [21], but there are conflicting results from another study [22].

To clarify the correlation between the expression and methylation of TIP30 and the prognosis of tumor patients, we conducted a meta-analysis to evaluate the influence of TIP30 on the overall survival, recurrence (disease) free survival, and clinicopathological features of malignant tumors.

## Materials and Methods

### Identification of eligible studies

Studies were searched for in PubMed and EMBASE without language limitations. The search time was from January 1997 to January 2016. The search terms used were as follows: “TIP30 OR CC3 OR HTATIP2” and “survival OR prognostic OR prognosis.” The strategy used both MeSH terms and free-text words to increase the searching sensitivity.

In addition to electronic databases, printed journals and relevant textbooks were manually searched in the libraries of the Beijing University of Chinese Medicine, Peking Union Medical College, and Guang’anmen Hospital. In addition, specialized experts in particular fields were consulted for necessary supplements.

Inclusion criteria were as follows: (1) patient-histologic diagnosis of malignant neoplasms; (2) TIP30 protein or mRNA in the primary tumor tissues was assessed by using an immunohistochemical or polymerase chain reaction (PCR) method; and (3) to assess the relationship between TIP30 and outcome variables and clinicopathological features at least one of the following needed to be reported: overall survival time, TNM clinical stage, lymph node metastasis, poor pathology grade, blood metastasis, or depth of tumor invasion.

Exclusion criteria were as follows: (1) reviews and single case reports; (2) studies referring to TIP30 but not to human cancer; and (3) lack of outcome variables and clinicopathological features between TIP30 and human cancer.

### Data extraction and management

Two independent reviewers (Y.Y. and Z.J.) extracted data from the eligible studies by using a standardized collection form. We recorded the details of the eligible studies including first author, characteristics of patients, publication year, pathology type, TIP30 assay methods, total cases, clinicopathological features, and outcomes. If there were discrepancies between the two reviewers a final consensus was reached after a discussion with H.Z. The hazard ratio was extracted directly if it was reported in the article or it was estimated from the Kaplan-Meier survival curve and the 5 year survival outcome events using the methods reported by Tierney [23].

### Methodological assessment

Methodological assessment of eligible studies was conducted using a quality scale for biological prognostic factors reported previously (S4 File)[24]. Two specialists (Q.G. and T.X.) who are experienced in clinical and basic experiments rated the studies. Disagreements between the two reviewers were discussed with B.H. before reaching a final consensus.

### Statistical analysis

The statistical analyses were performed using Review Manager (Revman) 5.3.5 software (Cochrane Community, London, United Kingdom) and STATA 14 software. The dichotomous data of the clinicopathological features were pooled using odds ratios (ORs) with 95% confidence intervals (CI). Hazard ratios (HRs) were pooled as inverse variance data with 95% CI. P < 0.05 was considered to indicate a statistically significant difference. An observed HR or OR > 1 implied a worse prognosis for the group that was TIP30 positive and was considered to be statistically significant if the 95% CI did not overlap 1. The heterogeneity of the included studies was evaluated by the χ2 and I^2^ tests, and P < 0.10 or I^2^ > 50% was defined as indicating heterogeneity. The fixed effect model was used for pooling homogeneous data and the random effect model was used for heterogeneous data. The sources of heterogeneity were investigated by subgroup analysis and meta-regression (using STATA 14 software) with p < 0.05 in meta-regression indicating a contribution of heterogeneity. Publication bias was evaluated by the symmetry of the funnel plot (Revman 5.3.5 software), Egger’s test, Begg’s test and the trim and fill method (R 3.3.1 software). When the funnel plot was visually asymmetric and p < 0.05 on Egger’s and Begg’s tests, significant bias was indicated. Sensitivity analysis was performed by reanalyzing the data using different statistical approaches.

## Results

### Included studies and characteristics

**Fig 1:**
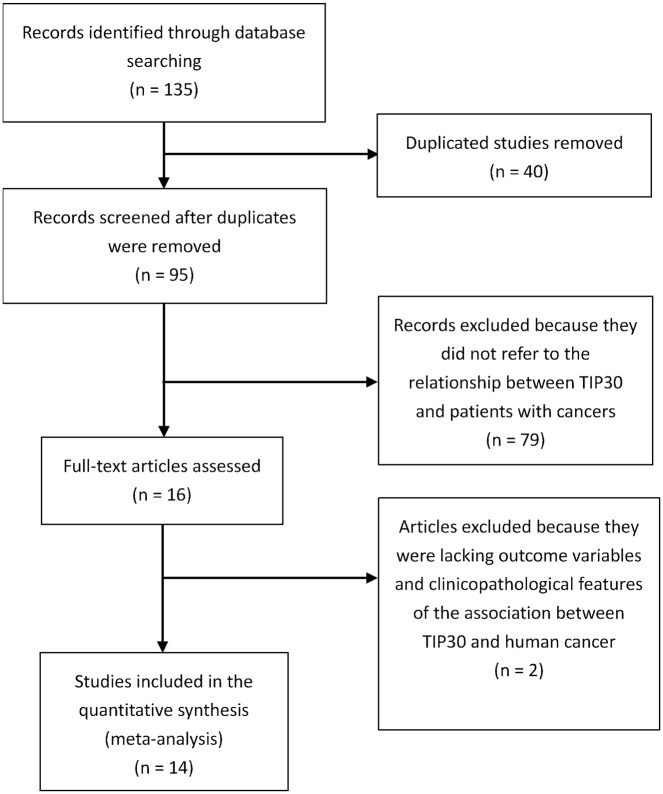
Flow diagram of the literature search process.

The initial search strategy identified 135 studies; 40 duplicated studies were removed, 79 studies were excluded because they did not refer to the relationship between TIP30 and patients with cancers, and 2 studies were excluded because they lacked relevant outcome variables and clinicopathological features associating TIP30 and human cancer (Fig 1). Finally, 14 studies [12– 22,25–27] with 1705 patients were included (shown in Table 1). Twelve articles reported the HR between overall survival time and TIP30. Six articles studied the relationship between TIP30 and disease free survival/recurrence free survival (DFS/RFS) of cancer patients. In addition to the expression of TIP30, two articles explored the HR between methylation of the TIP30 promoter and OS or DFS of cancer patients. TIP30 expression was detected by immunohistochemistry (IHC) and the methylation was detected by methylation-specific polymerase chain reaction (MS-PCR). The methodological assessment of eligible studies was conducted as described in Table 2.

**Table 1:** Characteristics of the included studies.

Study	Year	Region	Sample size (n)	No. of TIP30 high expression/promoter methylation(%)	Tumor type	TNM stage	Methods of TIP30 identification	Cut-off	Clinical pathological information	Outcome measures	Survival analysis
Bu F^[^^12^^]^	2014	China	137	68(49.6%)	Esophageal carcinoma	I-IV	IHC	≥20%	TNM clinical stage, Pathological grade, Lymph node metastasis, Distant metastases, Degree of invasion	OS^b^	Univariate
Chen J^[^^13^^]^	2015	China	105	49(46.7%)	Laryngeal carcinoma	I-IV	IHC	≥10%	TNM clinical stage, Pathological grade, Lymph node metastasis	OS, DFS	Multivariate
Dong X^[^^21^^]^	2015	China	Expression 50 Methylation 40	Expression 31(62%) Methylation 24(60%)	Glioma	I-IV	IHC/MS-PCR^a^	≥25%	TNM clinical stage	OS	Univariate
Guo S^[^^15^^]^	2013	China	106	54(50.9%)	Pancreatic ductal adenocarcinoma	I-III	IHC	≥25%	Pathological grade, Degree of invasion, Lymph node metastasis, Neural invasion	OS, RFS	Multivariate
Hu Y^[^^14^^]^	2015	China	92	50(%)	Glioma	I-IV	IHC	≥10%	TNM clinical stage	OS	Multivariate
Huang Q^[^^16^^]^	2011	China	112	50(44.6%)	Breast cancer	I-III	IHC	≥10%	TNM clinical stage, Lymph node metastasis	OS	Univariate
Li X^[^^17^^]^	2009	China	106	51(%)	Gastric cancer	I-IV	IHC	≥25%	Pathologic grade, TNM clinical stage	OS	Univariate
Liu B^[^^22^^]^	2008	China	52	Methylation 22(%)	Hepatocellular carcinoma	Unclear	MS-PCR^a^	Unclear	AFP level, HBV positive	OS, DFS	Univariate
Liu D^[^^18^^]^	2011	China	108	54(50%)	Gallbladder adenocarcinoma	Unclear	IHC	≥25%	Pathological grade, Lymph node metastasis, Local infiltration	OS	Multivariate
Wang W^[^^25^^]^	2014	China	297	149(%)	Hepatocellular carcinoma	I-IIIA	IHC	Unclear	TNM clinical stage, Pathological grade, Vascular invasion, Intrahepatic metastasis	OS, RFS	Multivariate
Zhu M^[^^26^^]^	2014	China	105	59(56.2%)	Laryngeal carcinoma	I-IV	IHC	≥25%	TNM clinical stage, Pathological grade, Lymph node metastasis, Degree of invasion	OS, RFS	Multivariate
Zhu M^[^^20^^]^	2015	China	151	92(60.9%)	Hepatocellular carcinoma	I-IV	IHC	≥25%	TNM clinical stage, Lymphovascular invasion, AFP, Hepatocirrhosis	OS, RFS	Multivariate
Tong X^[^^19^^]^	2009	China	197	125(%)	Lung cancer	I-IV	IHC	≥25%	TNM clinical stage, Pathological grade, Lymph node metastasis	-	-
Zhao J^[^^27^^]^	2006	China	87	45(52%)	Breast cancer	Unclear	IHC	≥1%	Vascular invasion, Lymph node metastasis, Tumor differentiation	-	-

^a^. MS-PCR: Methylation-specific polymerase chain reaction

^b^. OS: Overall survival; DFS: Disease-free survival; RFS: Recurrence-free survival.

**Table 2:** Quality assessment of the included studies.

	Scientific design	Laboratory methodology	Generalizability	Results analysis	Total score (%)
Bu F	8	9	5	4	59.09
Chen J	8	9	8	6	70.45
Dong X	8	10	5	5	63.64
Guo S	8	12	8	7	79.55
Huang Q	8	8	8	5	65.91
Li X	8	11	4	4	61.36
Liu B	8	10	4	5	64.29
Liu D	8	9	5	6	63.64
Wang W	8	10	8	7	75.00
Zhu M (2014)	8	11	5	7	70.45
Zhu M (2015)	8	8	5	7	63.64
Hu Y	8	9	8	6	70.45
Tong X	6	8	4	0	40.91
Zhao J	6	8	5	0	43.18

### Results of the meta-analysis

#### High expression of TIP30 in tumor tissues was associated with an improved prognosis of cancer patients

**Fig 2:**
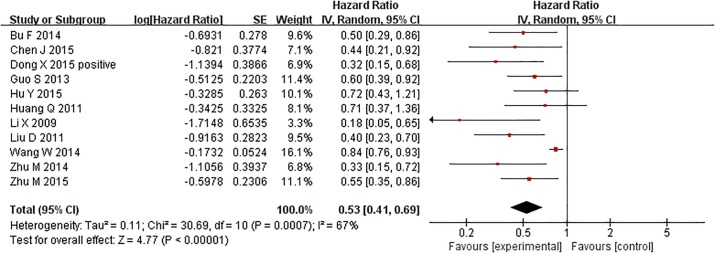
Forest plot of hazard ratios (HRs) of OS in the random-effect model. The HR of the overall survival time of TIP30-high expression cancer patients was compared with TIP30-low expression cancer patients. Each individual study is represented by a red square, and the pooled datasets are indicated by a diamond, representing the 95% confidence interval (CI) of each study. A HR < 1 implies a better survival for the cancer patients. The size of each study represents the weighting factor (1/standard error [SE]) assigned to it.

**Fig 3:**
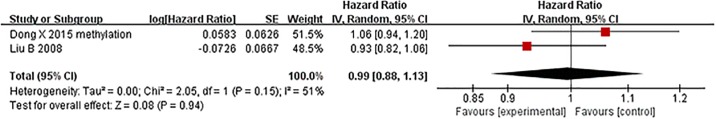
Forest plot of hazard ratios (HRs) of OS in the random-effect model. The HR of overall survival time of TIP30 promoter methylated cancer patients was compared with TIP30 promoter unmethylated cancer patients. A HR = 0.99 implies no significant differences in OS for methylation of the TIP30 promoter.

Eleven studies reported the overall survival (OS) of cancer patients and TIP30 expression in tumor tissues. A significant association was observed between TIP30 expression and OS. This suggests that high expression of TIP30 might be associated with a prolonged overall survival time of cancer patients (random effect model HR = 0.53, 95% CI: 0.41–0.69), but significant heterogeneity was detected among studies (Chi^2^ = 30.69, df = 10, p = 0.0007, I^2^ = 67%) (Fig 2). The same result was reached in the sensitivity analysis using a fixed effect model (HR = 0.75, 95% CI: 0.68–0.82) (Fig 2 and Figure A in S1 File). Two articles studied the relationship between methylation of the TIP30 promoter and OS. There were not significant differences in OS related to methylation of the TIP30 promoter (random effect model HR = 0.99, 95% CI: 0.88–1.13), but significant heterogeneity was detected among studies (Chi^2^ = 2.05, df = 1, p = 0.15, I^2^ = 51%) (Fig 3). The same result was reached in the sensitivity analysis using a fixed effect model (HR = 1.00, 95% CI: 0.91–1.09) (Fig 2 and Figure B in S1 File).

#### High expression of TIP30 in tumor tissues indicates a low disease progression rate in cancer patients

**Fig 4:**
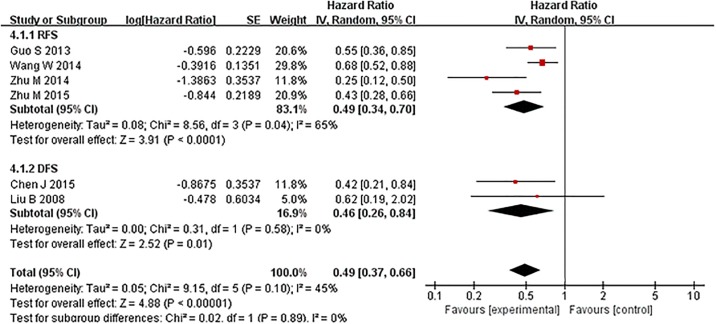
Forest plot of hazard ratios (HRs) of RFS/DFS in the random-effect model. The HR of recurrent free survival or disease free survival of TIP30-high expression cancer patients was compared with TIP30-low expression cancer patients. An HR<1 implied a better RFS/DFS for the cancer patients.

**Fig 5:**
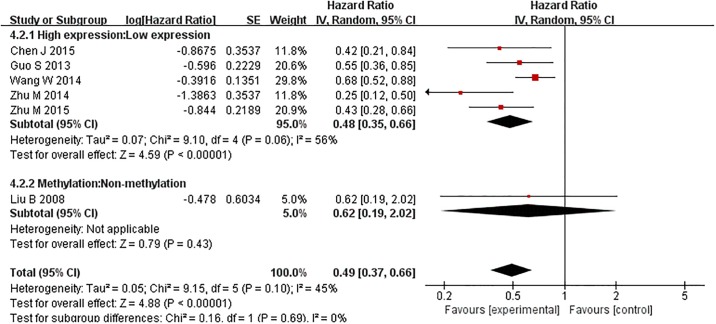
Forest plot of hazard ratios (HRs) of RFS/DFS in the random-effect model. The HR of RFS/DFS was compared within TIP30 expression and promoter methylation subgroups. An HR<1 implied a better RFS/DFS for the cancer patients.

Six studies reported the disease progress of cancer patients with TIP30 expression in tumor tissues. Four of them evaluated RFS and two of them studied DFS. Five studies evaluated the relationship between high expression of TIP30 and RFS/DFS in cancer patients, and one study evaluated the relationship between the methylation of the TIP30 promoter and DFS. A significant association was observed between TIP30 expression and disease progression. It has been suggested that high expression of TIP30 might be associated with a prolonged RFS and DFS for cancer patients (random model HR = 0.49, 95%CI: 0.37–0.66), but some heterogeneity was detected among the studies (Chi^2^ = 9.15, df = 5, p = 0.10, I^2^ = 45%) (Fig 4). The same result was reached in the sensitivity analysis using a fixed-effect model (HR = 0.54 with 95% CI: 0.45–0.65) (Fig 4 and Figure C in S1 File). The subgroup DFS and RFS analysis indicated that high expression of TIP30 could both prolong RFS (random effect model HR = 0.49 with 95% CI: 0.34–0.70) and DFS (random effect model HR = 0.46 with 95% CI: 0.26–0.84). However, the subgroup analysis of TIP30 high expression and promoter methylation indicated that methylation of the TIP30 promoter was not significantly associated with a prolonged DFS for tumor patients (random model HR = 0.62, 95% CI: 0.19–2.02) (Figs 4 and 5). Same results were reached in the sensitivity analysis using fixed effect model (Figures C and D in S1 File).

#### Subgroup Analyses

Because of the presence of heterogeneity, subgroup analyses were performed based on the tumor types, manuscript quality, survival analysis, cut-off of TIP30 identification, and number of patients (Table 3 and Figures E-I in S1 File).

**Table 3:** Results of the subgroup analysis of the included studies.

Study subgroups	No. of studies	No. of patients	Fixed	Pooled HR [95% CI]		P value	Heterogeneity		Meta-regression P value
				P value	Random		I^2^ (%)	P value	
Tumor types									0.012
glioma	2	142	0.56 [0.36, 0.85]	0.007	0.50 [0.23, 1.11]	0.09	67	0.08	
laryngeal carcinoma	2	210	0.38 [0.23, 0.65]	0.0004	0.38 [0.23, 0.65]	0.004	0	0.60	
esophageal carcinoma	1	137	0.50 [0.29, 0.86]	0.01	0.50 [0.29, 0.86]	0.01	Not applicable		
pancreatic carcinoma	1	106	0.60 [0.39, 0.92]	0.02	0.60 [0.39, 0.92]	0.02	Not applicable		
breast cancer	1	112	0.71 [0.37, 1.36]	0.30	0.71 [0.37, 1.36]	0.30	Not applicable		
gastric cancer	1	106	0.18 [0.05, 0.65]	0.009	0.18 [0.05, 0.65]	0.009	Not applicable		
hepatocellular cancer	2	203	0.82 [0.75, 0.91]	0.0001	0.72 [0.48, 1.08]	0.11	69	0.07	
gallbladder cancer	1	108	0.40 [0.23, 0.70]	0.001	0.40 [0.23, 0.70]	0.001	Not applicable		
Quality score (%)									0.895
≥65	6	817	0.80 [0.73, 0.88]	<0.00001	0.65 [0.50, 0.85]	0.002	52	0.06	
<65	5	604	0.44 [0.34, 0.58]	<0.00001	0.44 [0.34, 0.58]	<0.00001	0	0.43	
Survival analysis									0.043
Univariate	5	556	0.50 [0.38, 0.65]	<0.00001	0.49 [0.35, 0.67]	<0.0001	21	0.28	
Multivariate	6	865	0.79 [0.71, 0.86]	<0.00001	0.58 [0.41, 0.80]	0.0009	69	0.006	
Cut-off									0.003
≥25%	6	626	0.46 [0.36, 0.59]	<0.00001	0.45 [0.34, 0.59]	<0.00001	16	0.31	
Other values	5	743	0.81 [0.74, 0.89]	<0.0001	0.70 [0.55, 0.90]	0.005	38	0.17	
Sample size (n)									0.960
≥110	4	697	0.81 [0.73, 0.89]	<0.0001	0.68 [0.51, 0.91]	0.01	54	0.09	
<110	7	724	0.48 [0.38, 0.61]	<0.00001	0.46 [0.35, 0.61]	<0.00001	26	0.23	

We detected a significant association between high expression of TIP30 and a good OS of patients with esophageal carcinoma (HR = 0.50, 95% CI: 0.29–0.86), laryngeal carcinoma (HR = 0.38, 95% CI: 0.23–0.65), pancreatic adenocarcinoma (HR = 0.60, 95% CI: 0.39–0.92), gastric cancer (HR = 0.18, 95% CI: 0.05–0.65), and gallbladder adenocarcinoma (HR = 0.40, 95% CI: 0.23–0.70). TIP30 was also associated with a prolonged OS of patients with hepatocellular carcinoma (HR = 0.72, 95% CI: 0.48–1.08), glioma (HR = 0.50, 95% CI: 0.23–1.11), and breast cancer (HR = 0.71, 95% CI: 0.37–1.36), but not significantly. Because significant heterogeneity was detected among the glioma and hepatocellular carcinoma subgroups, we adopted the results of the random effect model.

In addition, the quality of the study (rated as more than 65% or less than 65%) did not influence the results of the estimated HR (HR 0.65, 95% CI: 0.50–0.85 and HR 0.44, 95% CI: 0.34–0.58 respectively). An analysis of the subgroups using different cut-off values of TIP30 showed a good OS for the cut-off ≥ 25 and other values (HR 0.46, 95% CI: 0.36–0.59 and HR 0.81, 95% CI: 0.74–0.89, respectively). The survival analysis did not affect the pooled HR; HRs were 0.50 (95% CI: 0.38–0.65) and 0.58 (95% CI: 0.41–0.80), respectively, in univariate and multivariate subgroups. Moreover, high expression of TIP30 was closely associated with a good OS of tumor patients in the subgroups based on sample size (more than 110 or less than 110), and the pooled HRs were 0.68 (95% CI: 0.51–0.91) and 0.48 (95% CI: 0.38–0.61), respectively. Because significant heterogeneity was detected among studies with a quality score ≥ 65, multivariate survival analysis, and sample size ≥ 110 subgroups, results of the random effect model were adopted. The meta-regressions indicated that tumor types, survival analysis and cut-off value might contribute the most heterogeneity (p values were 0.012, 0.043 and 0.003 respectively).

#### Associations between TIP30 protein and the clinicopathological characteristics of tumor patients

***Lack of TIP30 expression could lead to late TNM clinic stage and lymph node metastasis*:** The prognostic significance of low expression of TIP30 in the TNM clinical stage was evaluated in 10 studies with 1351 patients. The results showed that low expression of TIP30 could lead to a high TNM clinical stage (III or IV clinical stage) in tumor patients (fixed effect model: OR = 2.10, 95% CI: 1.68–2.62) without significant heterogeneity. In the analysis of eight studies with 959 patients, low expression of TIP30 was significantly associated with lymph node metastasis in tumor patients (random effect model: OR = 3.90, 95% CI: 2.21–6.89). Significant heterogeneity was detected but the sensitive analysis showed that the OR was not influenced by using different statistical approaches (fixed effect model: OR = 3.51, 95% CI: 2.67–4.63) (Table 4 and Figures J and K in S1 File).

**Table 4:** Meta-analysis of TIP30 and the clinical and pathological features of patients with tumor.

Clinical and pathological features	No. of studies	No. of patients	Pooled OR [95% CI]				Heterogeneity	
			Fixed	P value	Random	P value	I^2^ (%)	P value
III/IV clinical stage	10	1351	2.10 [1.68, 2.62]	<0.00001	2.17 [1.62, 2.89]	<0.00001	34	0.13
Lymph node metastasis	8	959	3.51 [2.67, 4.63]	<0.00001	3.90 [2.21, 6.89]	<0.00001	74	0.0004
Poor differentiation	8	1142	1.48 [1.14, 1.91]	0.003	1.49 [0.87, 2.56]	0.14	73	0.0005
Vascular tumor thrombus	4	641	1.96 [1.39, 2.76]	0.0001	2.04 [0.57, 7.33]	0.27	88	<0.0001
T3/4 invasion	4	456	1.60 [1.08, 2.37]	0.02	1.62 [0.80, 3.29]	0.18	67	0.03

***The role of TIP30 in poor differentiation*, *vascular tumor thrombus*, *and deep invasion of tumor patients is unclear*:** We evaluated the relationship between low expression of TIP30 and poor differentiation, vascular tumor thrombus, and deep invasion. There was a discrepancy between the fixed effect and random effect models. Because significant heterogeneity was detected, the random effect model was used and the results showed that low expression of TIP30 was associated with poorer pathological differentiation (random effect model: OR = 1.49, 95% CI: 0.87–2.56), vascular tumor thrombus (random effect model: OR = 2.04, 95% CI: 0.57–7.33), and deep tumor invasion (random effect model: OR = 1.62, 95% CI: 0.80–3.29), but not significantly (Table 4 and Figures L-N in S1 File).

### Publication bias

**Fig 6:**
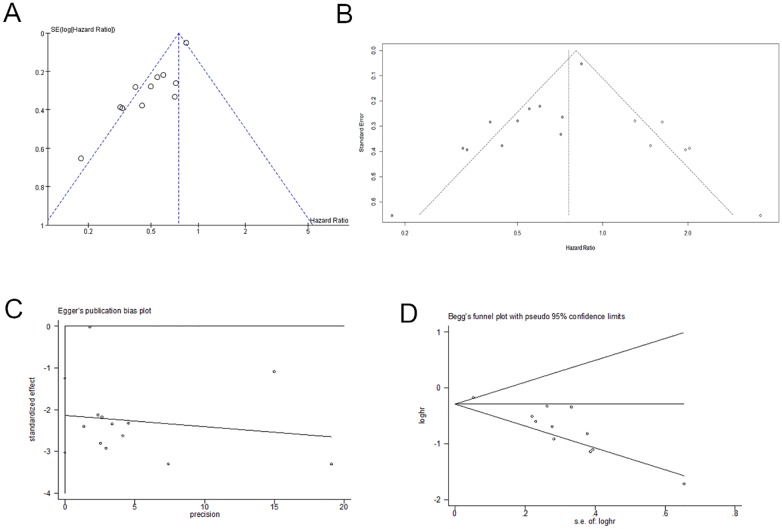
Texts of publication bias. The Test is based on a linear regression of the standard normal deviate against its precision. In our analysis, we used the inverse of the standard error as the independent variable and the standardized estimate of the size effect (log HR upon its standard error) as the dependent variable. The estimate of the effect is considered biased if the intercept is significantly different from zero. Figures: A. Funnel plot; B. Trim and fill method; C. Egger’s text; D. Begg’s test.

The possibility of publication bias was evaluated by visual inspection of funnel plots, Egger’s test and Begg’s test using STATA 14 software with p < 0.05 indicating significant bias. The test results are shown in Fig 6 and the S2 File. The funnel plot was visually asymmetric and Egger's test and Begg’s test both suggested that publication bias may have a significant influence on the results of the HR for OS for tumor patients (p < 0.001). The same result was obtained in using the trim and fill method.

## Discussion

TIP30 was first identified and characterized as a candidate tumor-suppressor gene in 1997 [10]. Recently, the tumor suppressor status of TIP30 has been fully established. Studies showed that TIP30 is involved in the control of cell apoptosis, growth, metastasis, angiogenesis, DNA repair, and tumor cell metabolism [28]. For instance, TIP30 has a significant effect on DNA repair after ultraviolet light and oxidant exposure, and can regulate the metabolic adaptation of tumor cells to glucose limitations [29,30]. Moreover, TIP30 regulates p53 at the protein level by directly binding to it, and it might regulate the *BAX* gene partly through directly binding to p53 protein, which sensitizes cells to apoptosis by involving a p53 apoptosis signal transduction pathway [31,32]. Researchers also showed that TIP30 could inhibit snail-mediated epithelial-mesenchymal transition (EMT) and tumor-initiating properties in hepatocellular carcinoma and suppress TGF-β1-induced EMT via AKT/β-catenin signaling in esophageal carcinoma[12,20].

Based on its tumor suppressing effects in previous research, we conducted this meta-analysis to evaluate the clinical usefulness of TIP30 expression in diagnosis and treatment. It has been suggested that high expression of TIP30 is associated with prolonged overall survival time and disease free survival time for tumor patients. The DNA methylation status of TIP30 gene may be associated with this and affect the sensitivity of tumor cells to chemotherapy [33]. In our study, there was no significant relationship between the promotor methylation of TIP30 and the OS or DFS. This might be confusing since previous studies have suggested that methylation of TIP30 could down-regulate its expression [21]. Still, other epigenetic alterations such as gene mutations or loss of heterozygosity might also affect the expression of TIP30. Hence, there might be no statistically significant correlation between the down-regulation of TIP30 and Tip30 promoter methylation. Thus, there could be no significant relationship between the promotor methylation of TIP30 and the prognosis of tumor patients. Furthermore, in consideration of the small sample size, conclusions relating to the TIP30 promotor methylation should be interpreted with discretion. In subgroup analysis, high expression of TIP30 had a significant association with longer OS for esophageal carcinoma, laryngeal carcinoma, pancreatic adenocarcinoma, gastric cancer, and gallbladder adenocarcinoma patients. TIP30 was also associated with a prolonged OS for lung cancer, hepatocellular carcinoma, glioma, and breast cancer patients, but not significantly.

Because there was a significant heterogeneity in the OS evaluation, we conducted meta regressions among the subgroups. Our results suggest that tumor types, survival analysis and cut-off values might contribute the most to the heterogeneity. To further explore the diagnostic value of TIP30, we evaluated the relationship between TIP30 and the clinical and pathological features of tumor patients. Our results suggest that a lack of TIP30 expression is associated with lymph node metastasis and an advanced clinical stage of tumor patients. Although TIP30 is involved in inhibiting tumorigenesis, angiogenesis, and metastasis in basic research, our results for these associations were inconsistent with high heterogeneity. The random effect model was used and there were no significant relationships between TIP30 and poor differentiation, more vascular tumor thrombus, or high grade tumor invasion.

There were some limitations in our study. First, all studies included in our evaluation were conducted in China; hence, the conclusions can only be carefully applied to China or the East Asia area, not worldwide. Second, there was some heterogeneity among the eligible studies, the types of tumors were varied, and their quality scores were diverse. These factors might have led to significant publication bias and influenced our evaluation.

In conclusion, the high expression of TIP30 is a good prognostic indicator for tumor patients, associated with a prolonged OS and DFS/RFS. Low or lack of expression of TIP30 is associated with a poor prognosis of tumor patients and with lymph node metastasis and late tumor clinical stage. However, the association between methylation of the TIP30 promoter and tumor prognosis is unclear. Furthermore, drugs promoting the expression of TIP30 should be studied and used in cancer treatment. Clinical studies with large samples are needed to evaluate the relationship between TIP30 protein, TIP30 promoter methylation, and tumor patients worldwide, and standardized protocols should be adopted in future studies. We suggest that future studies should include all of the following information, such as the patients’ TNM stages, TNM clinical stages and pathological types, and at least one outcome variables such as overall survival time and disease free survival should be observed. And the methods of TIP30 identification should be described in details, and the cut-off value about TIP30 Methylation should be unified.” We also suggest researchers should follow the REMARK recommendations [34], which provides the rationale and illustrative examples of good reporting and helps authors ensure that reports of their tumor marker studies contain the information that readers need.

## Supporting Information

S1 File Forest plots and results of the meta-analysis. (DOCX)

S2 File Publication bias of the meta-analysis. (DOC)

S3 File PRISMA Checklist for the meta-analysis. (DOC)

S4 File Quality scale for biological prognostic factors. (DOC)
