# Supplementary material for: Tat-Interacting Protein 30 (TIP30) Expression Serves as a New Biomarker for Tumor Prognosis: A Systematic Review and Meta-Analysis
Source: PLoS One. 2016 Dec 30;11(12):e0168408. doi: 10.1371/journal.pone.0168408 (PMC5201241; doi:10.1371/journal.pone.0168408)
Supplement: S1 File — (DOCX) [file pone.0168408.s001.docx]

**S1 file. Forest plots and results of mate-analysis**

**Forest plots of hazard ratios**
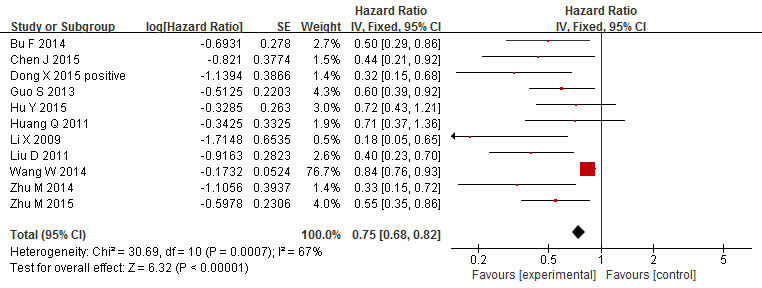


**Fig. 1. Forest plot of hazard ratios (HRs) of OS in the fixed-effects model.** The HR of the overall survival time of TIP30-high expression cancer patients was compared with TIP30-low expression cancer patients. Each individual study is represented by a red square, and the pooled datasets are indicated by a diamond, representing the 95% conﬁdence interval (CI) of each study. A HR < 1 implies a better survival for the cancer patients. The size of each study represents the weighting factor (1/standard error [SE]) assigned to it.


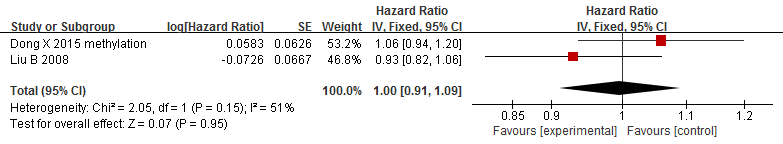


**Fig. 2. Forest plot of hazard ratios (HRs) of OS in the fixed-effects model.** The HR of overall survival time of TIP30 promoter methylated cancer patients was compared with TIP30 promoter unmethylated cancer patients. Each individual study is represented by a red square, and the pooled datasets are indicated by a diamond, representing the 95% CI of each study. A HR = 1.00 implies no significant differences in OS for methylation of the TIP30 promoter. The size of each study represents the weighting factor (1/standard error [SE]) assigned to it.


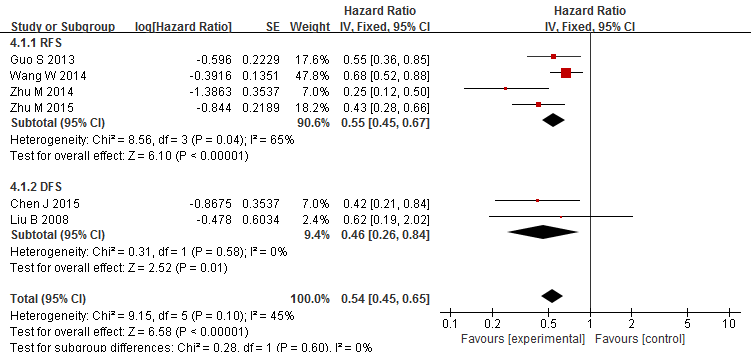


**Fig. 3. Forest plot of hazard ratios (HRs) of RFS/DFS in the fixed-effect model.** The HR of recurrent free survival or disease free survival of TIP30-high expression cancer patients was compared with TIP30-low expression cancer patients. Each individual study is represented by the red square, and the pooled datasets are indicated by the diamond, representing the 95% conﬁdence interval (CI) of each study. An HR<1 implied a better RFS/DFS for the cancer patients. The size of each study represents the weighting factor (1/standard error [SE]) assigned to it.


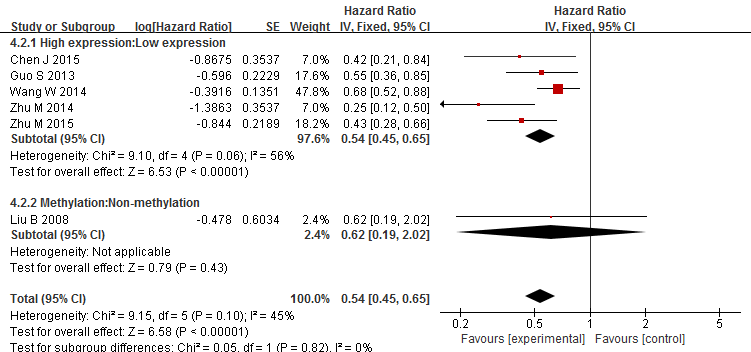


**Fig. 4. Forest plot of hazard ratios (HRs) of RFS/DFS in the fixed-effect model.** The HR of RFS/DFS was compared within TIP30 expression and promoter methylation subgroups. Each individual study is represented by the red square, and the pooled datasets are indicated by the diamond, representing the 95% conﬁdence interval (CI) of each study. An HR<1 implied a better RFS/DFS for the cancer patients. The size of each study represents the weighting factor (1/standard error [SE]) assigned to it.

**Subgroup Analyses of HRs**


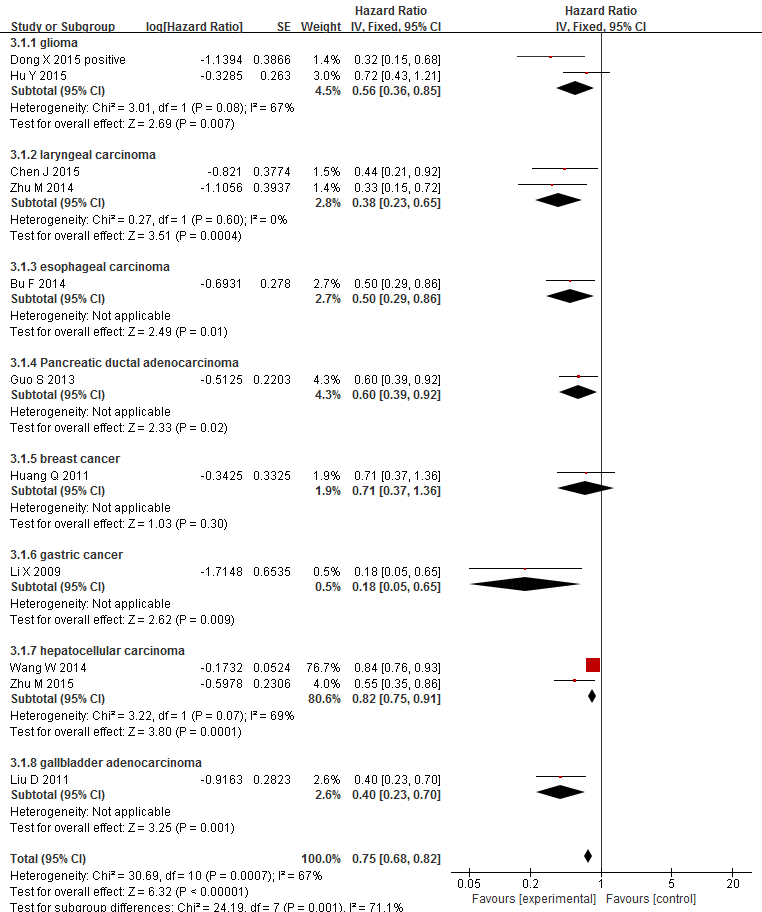


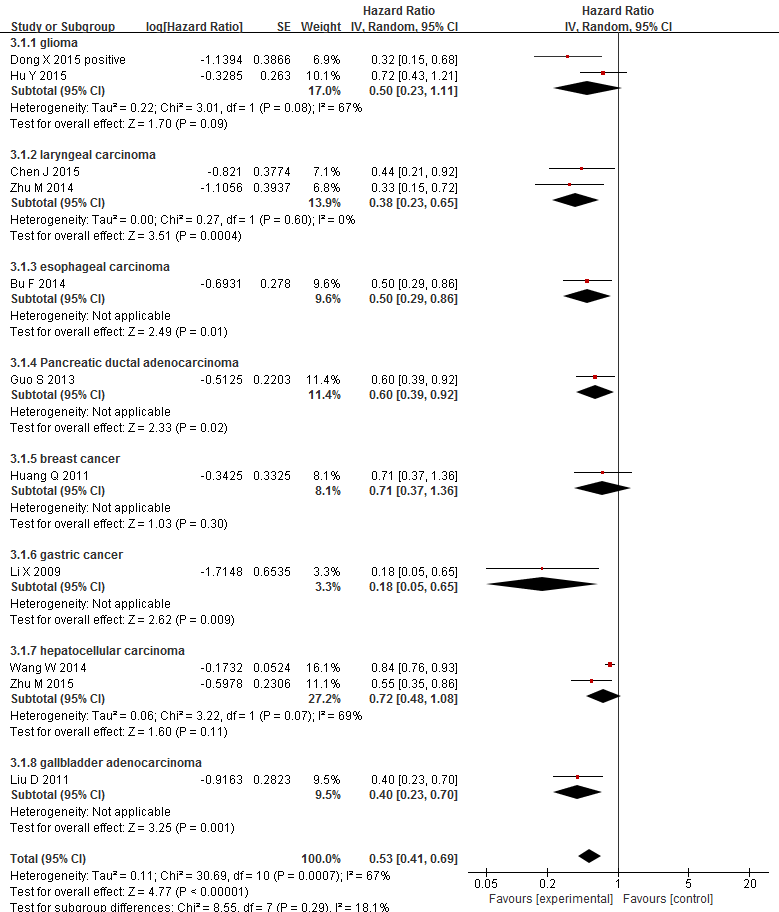


**Fig.5 Forrest plots of hazard ratio in fix/random effect model of subgroup analysis of tumor types.** The HR of the overall survival time of TIP30-high expression cancer patients was compared with TIP30-low expression cancer patients. Each individual study is represented by a red square, and the pooled datasets are indicated by a diamond, representing the 95% conﬁdence interval (CI) of each study. A HR < 1 implies a better survival for the cancer patients. The size of each study represents the weighting factor (1/standard error [SE]) assigned to it.


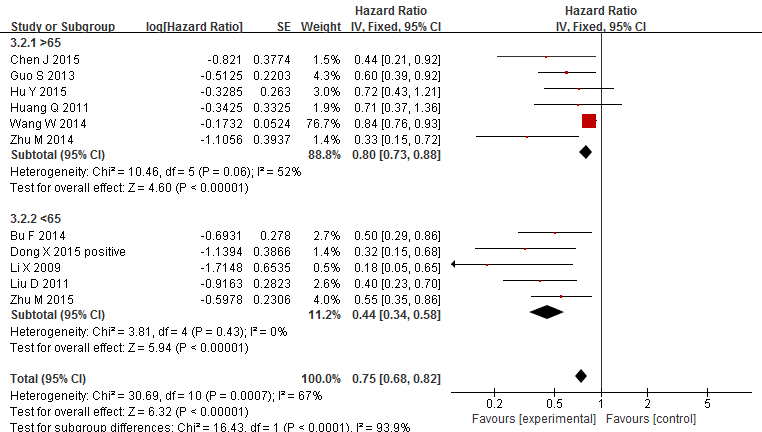


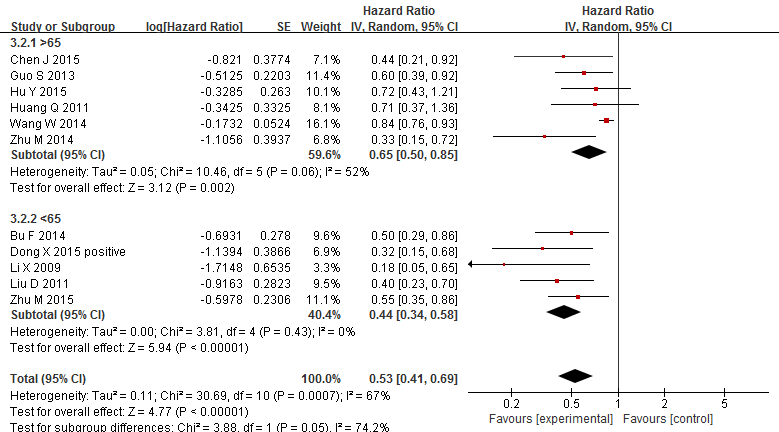


**Fig.6 Forrest plots of hazard ratio in fix/random effect model of subgroup analysis of quality score.** The HR of the overall survival time of TIP30-high expression cancer patients was compared with TIP30-low expression cancer patients. Each individual study is represented by a red square, and the pooled datasets are indicated by a diamond, representing the 95% conﬁdence interval (CI) of each study. A HR < 1 implies a better survival for the cancer patients. The size of each study represents the weighting factor (1/standard error [SE]) assigned to it.


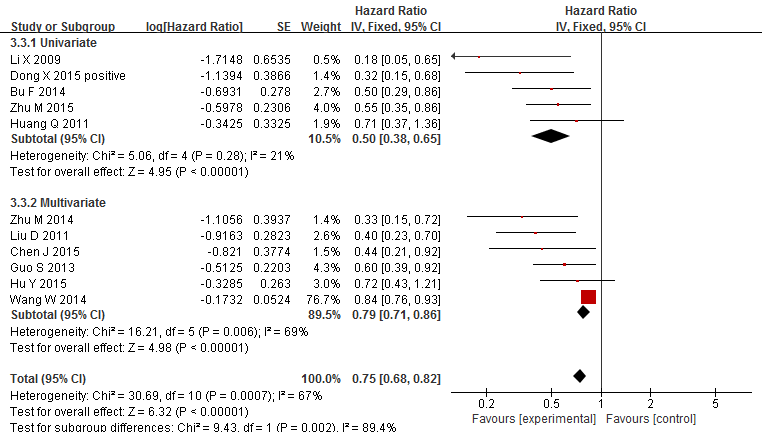


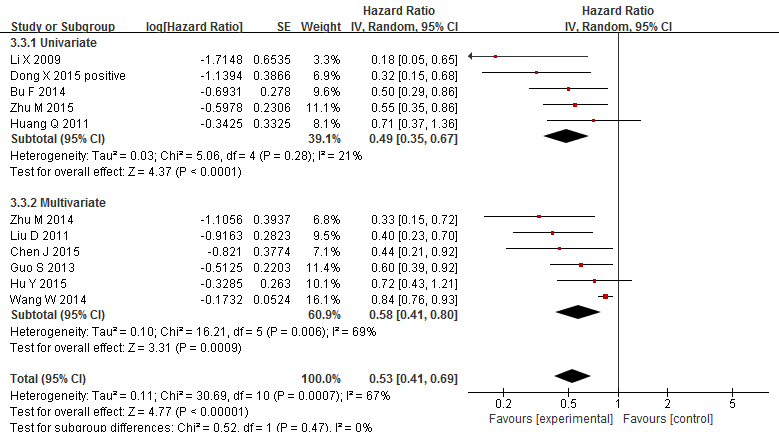


**Fig.7 Forrest plots of hazard ratio in fix/random effect model of subgroup analysis of survival analysis.** The HR of the overall survival time of TIP30-high expression cancer patients was compared with TIP30-low expression cancer patients. Each individual study is represented by a red square, and the pooled datasets are indicated by a diamond, representing the 95% conﬁdence interval (CI) of each study. A HR < 1 implies a better survival for the cancer patients. The size of each study represents the weighting factor (1/standard error [SE]) assigned to it.


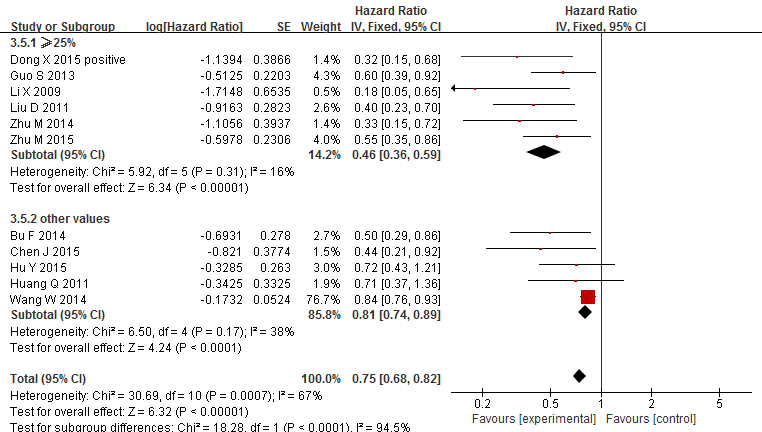


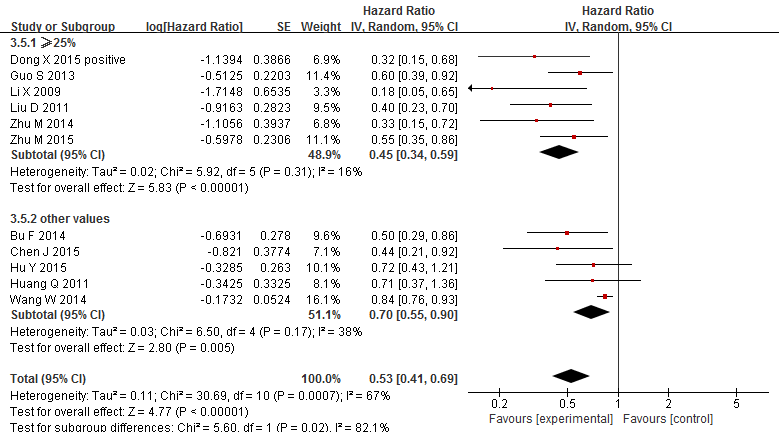


**Fig.8 Forrest plots of hazard ratio in fix/random effect model of subgroup analysis of cut-off values.** The HR of the overall survival time of TIP30-high expression cancer patients was compared with TIP30-low expression cancer patients. Each individual study is represented by a red square, and the pooled datasets are indicated by a diamond, representing the 95% conﬁdence interval (CI) of each study. A HR < 1 implies a better survival for the cancer patients. The size of each study represents the weighting factor (1/standard error [SE]) assigned to it.


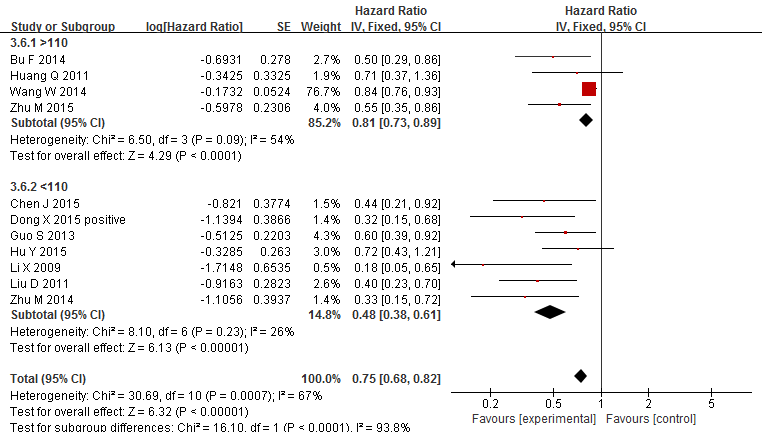


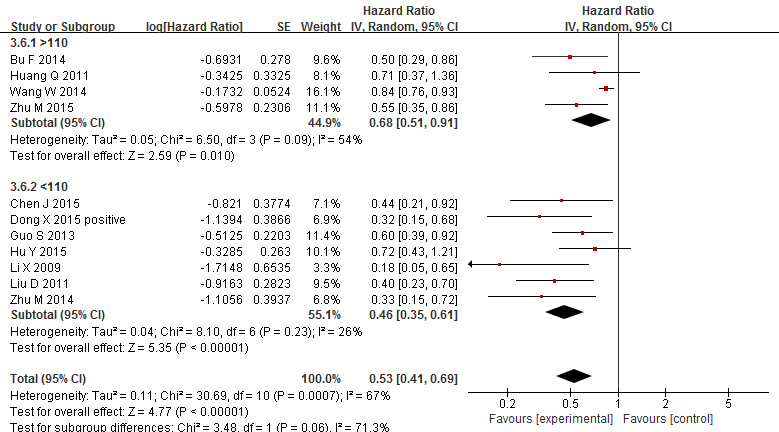


**Fig.9 Forrest plots of hazard ratio in fix/random effect model of subgroup analysis of sample size.** The HR of the overall survival time of TIP30-high expression cancer patients was compared with TIP30-low expression cancer patients. Each individual study is represented by a red square, and the pooled datasets are indicated by a diamond, representing the 95% conﬁdence interval (CI) of each study. A HR < 1 implies a better survival for the cancer patients. The size of each study represents the weighting factor (1/standard error [SE]) assigned to it.

**Associations between TIP30 protein and the clinicopathological characteristics of tumor patients**


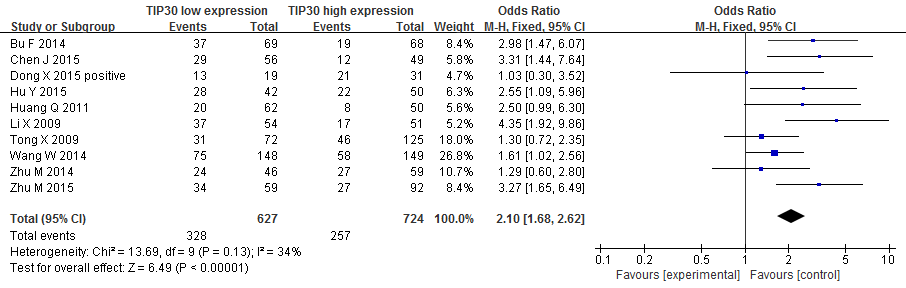


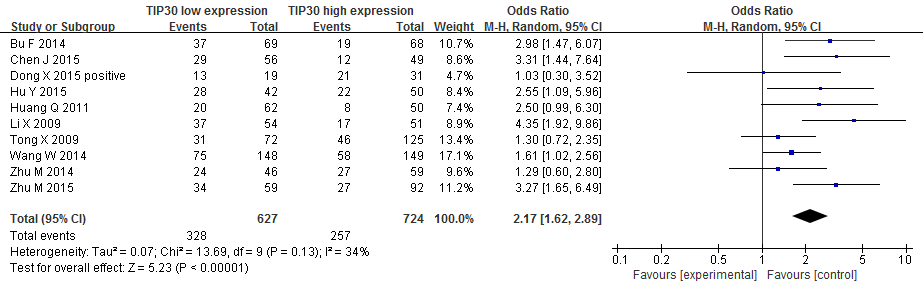


**Fig.10 Forrest plots of odds ratio in fix/random effect model of analysis of III/IV clinical stage.** The OR of the III/IV clinical stage of TIP30-high expression cancer patients was compared with TIP30-low expression cancer patients. Each individual study is represented by a blue square, and the pooled datasets are indicated by a diamond, representing the 95% conﬁdence interval (CI) of each study. An OR >1 implies a higher rate for the III/IV clinical stage cancer patients. The size of each study represents the weighting factor (1/standard error [SE]) assigned to it.


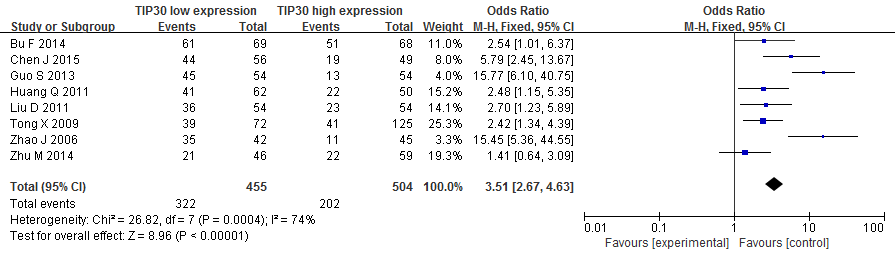


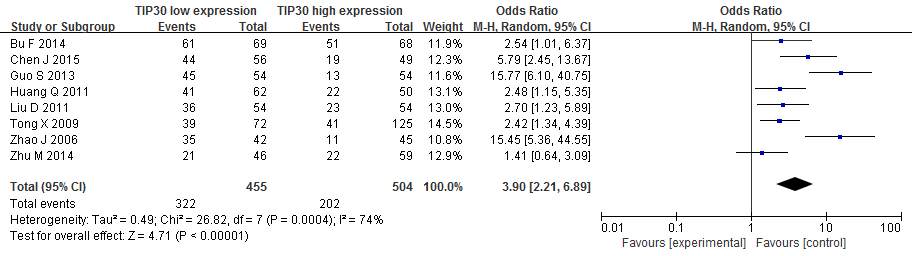


**Fig.11 Forrest plots of odds ratio in fix/random effect model of analysis of lymph node metastasis.** The OR of the lymph node metastasis of TIP30-high expression cancer patients was compared with TIP30-low expression cancer patients. Each individual study is represented by a blue square, and the pooled datasets are indicated by a diamond, representing the 95% conﬁdence interval (CI) of each study. An OR >1 implies a higher rate for the lymph node metastasis. The size of each study represents the weighting factor (1/standard error [SE]) assigned to it.


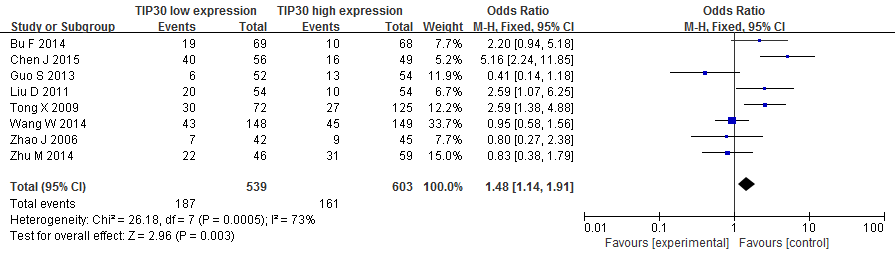


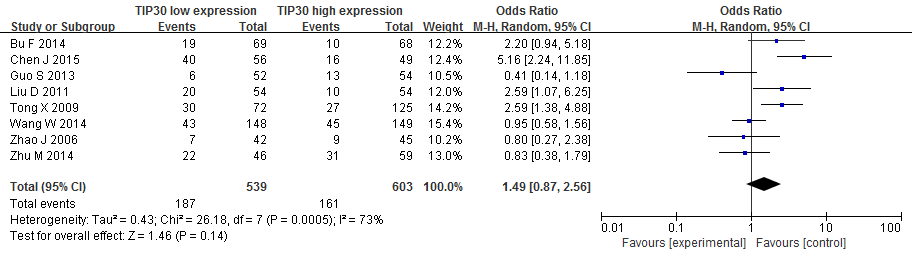


**Fig.12 Forrest plots of odds ratio in fix/random effect model of analysis of poor differentiation.** The OR of the poor differentiation of TIP30-high expression cancer patients was compared with TIP30-low expression cancer patients. Each individual study is represented by a blue square, and the pooled datasets are indicated by a diamond, representing the 95% conﬁdence interval (CI) of each study. An OR >1 implies a higher rate for the poor differentiation. The size of each study represents the weighting factor (1/standard error [SE]) assigned to it.


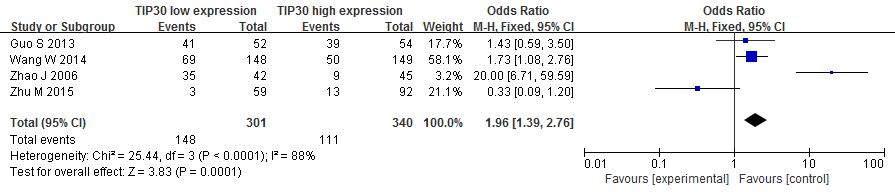


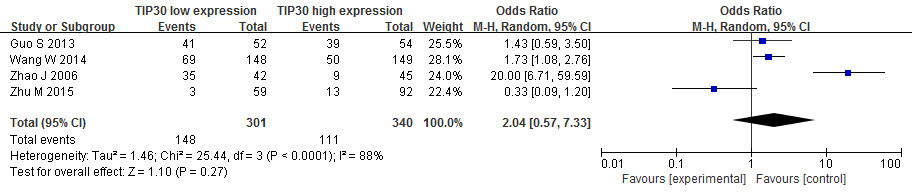


**Fig.13 Forrest plots of odds ratio in fix/random effect model of analysis of vascular tumor thrombus.** The OR of the vascular tumor thrombus of TIP30-high expression cancer patients was compared with TIP30-low expression cancer patients. Each individual study is represented by a blue square, and the pooled datasets are indicated by a diamond, representing the 95% conﬁdence interval (CI) of each study. An OR >1 implies a higher rate for the vascular tumor thrombus. The size of each study represents the weighting factor (1/standard error [SE]) assigned to it.


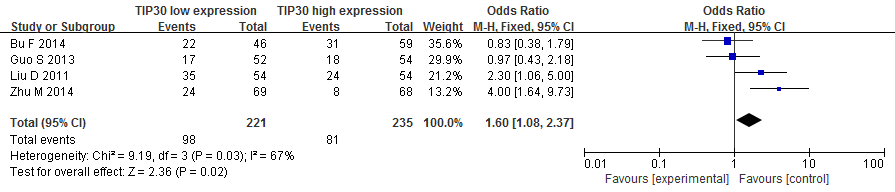


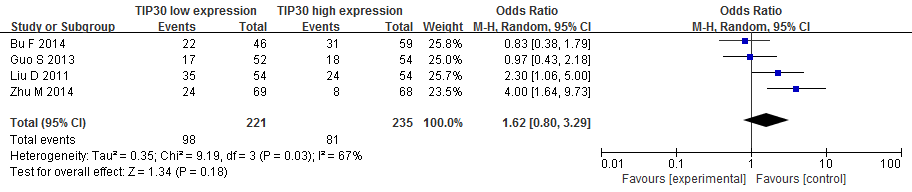


**Fig.14 Forrest plots of odds ratio in fix/random effect model of analysis of T3/4 invasion.** The OR of the T3/4 invasion of TIP30-high expression cancer patients was compared with TIP30-low expression cancer patients. Each individual study is represented by a blue square, and the pooled datasets are indicated by a diamond, representing the 95% conﬁdence interval (CI) of each study. An OR >1 implies a higher rate for the T3/4 invasion. The size of each study represents the weighting factor (1/standard error [SE]) assigned to it.
