# Supplementary material for: Tat-Interacting Protein 30 (TIP30) Expression Serves as a New Biomarker for Tumor Prognosis: A Systematic Review and Meta-Analysis
Source: PLoS One. 2016 Dec 30;11(12):e0168408. doi: 10.1371/journal.pone.0168408 (PMC5201241; doi:10.1371/journal.pone.0168408)

**S2 file. Publication bias**

HR

Tests for Publication Bias

Begg's Test

adj. Kendall's Score (P-Q) = -29

Std. Dev. of Score = 12.85

Number of Studies = 11

z = -2.26

Pr > |z| = 0.024

z = 2.18 (continuity corrected)

Pr > |z| = 0.029 (continuity corrected)

Egger's test

------------------------------------------------------------------------------

Std_Eff | Coef. Std. Err. t P>|t| [95% Conf. Interval]

-------------+----------------------------------------------------------------

slope | -.0547815 .0466242 -1.17 0.270 -.1602528 .0506897

bias | -2.18104 .3064142 -7.12 0.000 -2.874197 -1.487883

Test of trim and fill method

Meta-analysis

| Pooled 95% CI Asymptotic No. of

Method | Est Lower Upper z_value p_value studies

-------+----------------------------------------------------

Fixed | -0.288 -0.376 -0.199 -6.352 0.000 11

Random | -0.635 -0.896 -0.374 -4.774 0.000

Test for heterogeneity: Q= 30.649 on 10 degrees of freedom (p= 0.001)

Moment-based estimate of between studies variance = 0.107

Trimming estimator: Linear

Meta-analysis type: Random-effects model

iteration | estimate Tn # to trim diff

----------+--------------------------------------

1 | -0.635 25 0 66

2 | -0.635 25 0 0

Note: no trimming performed; data unchanged

Filled

Meta-analysis (exponential form)

> install.packages("meta")

将程序包安装入‘C:/Users/jzc/Documents/R/win-library/3.3’

(因为‘lib’没有被指定)

--- 在此連線階段时请选用CRAN的鏡子 ---

试开URL’https://mirrors.tuna.tsinghua.edu.cn/CRAN/bin/windows/contrib/3.3/meta_4.6-0.zip'

Content type 'application/zip' length 525974 bytes (513 KB)

downloaded 513 KB

程序包‘meta’打开成功，MD5和检查也通过

下载的二进制程序包在

C:\Users\jzc\AppData\Local\Temp\Rtmpg3nN6h\downloaded_packages里

> rhr=data.frame()

> fix(rhr)

> rhr

study loghr SEloghr hr ll ul

1 1 -0.6931 0.2780 0.50 0.29 0.86

2 2 -0.8210 0.3774 0.44 0.21 0.92

3 3 -1.1394 0.3866 0.32 0.15 0.68

4 4 -0.5125 0.2203 0.60 0.39 0.92

5 5 -0.3285 0.2630 0.72 0.43 1.21

6 6 -0.3425 0.3325 0.71 0.37 1.36

7 7 -1.7148 0.6535 0.18 0.05 0.65

8 8 -0.9163 0.2823 0.40 0.23 0.70

9 9 -0.1732 0.0524 0.84 0.76 0.93

10 10 -1.1056 0.3937 0.33 0.15 0.72

11 11 -0.5978 0.2306 0.55 0.35 0.86

> library("meta")

Loading 'meta' package (version 4.6-0).

Type 'help("meta-package")' for a brief overview.

Warning message:

程辑包‘meta’是用R版本3.3.2 来建造的

> metarhr= metagen(loghr, SEloghr, studlab=study, data=rhr, sm="HR", comb.random=TRUE)

> metarhr

HR 95%-CI %W(fixed) %W(random)

1 0.5000 [0.2900; 0.8622] 2.7 9.6

2 0.4400 [0.2100; 0.9219] 1.5 7.1

3 0.3200 [0.1500; 0.6827] 1.4 6.9

4 0.5990 [0.3890; 0.9225] 4.3 11.4

5 0.7200 [0.4300; 1.2056] 3.0 10.1

6 0.7100 [0.3700; 1.3623] 1.9 8.1

7 0.1800 [0.0500; 0.6479] 0.5 3.3

8 0.4000 [0.2300; 0.6956] 2.6 9.5

9 0.8410 [0.7589; 0.9319] 76.7 16.1

10 0.3310 [0.1530; 0.7161] 1.4 6.8

11 0.5500 [0.3500; 0.8643] 4.0 11.1

Number of studies combined: k = 11

HR 95%-CI z p-value

Fixed effect model 0.7483 [0.6840; 0.8188] -6.32 < 0.0001

Random effects model 0.5294 [0.4077; 0.6875] -4.77 < 0.0001

Quantifying heterogeneity:

tau^2 = 0.1074; H = 1.75 [1.28; 2.40]; I^2 = 67.4% [38.7%; 82.7%]

Test of heterogeneity:

Q d.f. p-value

30.69 10 0.0007

Details on meta-analytical method:

- Inverse variance method

- DerSimonian-Laird estimator for tau^2

> tf1 = trimfill (metarhr, comb.random=TRUE)

> tf1

HR 95%-CI %W(random)

1 0.5000 [0.2900; 0.8622] 6.6

2 0.4400 [0.2100; 0.9219] 5.3

3 0.3200 [0.1500; 0.6827] 5.1

4 0.5990 [0.3890; 0.9225] 7.5

5 0.7200 [0.4300; 1.2056] 6.9

6 0.7100 [0.3700; 1.3623] 5.9

7 0.1800 [0.0500; 0.6479] 2.7

8 0.4000 [0.2300; 0.6956] 6.6

9 0.8410 [0.7589; 0.9319] 9.5

10 0.3310 [0.1530; 0.7161] 5.1

11 0.5500 [0.3500; 0.8643] 7.4

Filled: 1 1.2955 [0.7513; 2.2339] 6.6

Filled: 2 1.4722 [0.7026; 3.0848] 5.3

Filled: 8 1.6195 [0.9313; 2.8162] 6.6

Filled: 10 1.9570 [0.9046; 4.2335] 5.1

Filled: 3 2.0242 [0.9488; 4.3185] 5.1

Filled: 7 3.5988 [0.9998; 12.9543] 2.7

Number of studies combined: k = 17 (with 6 added studies)

HR 95%-CI z p-value

Random effects model 0.7571 [0.5892; 0.9729] -2.17 0.0296

Quantifying heterogeneity:

tau^2 = 0.1689; H = 1.95 [1.54; 2.48]; I^2 = 73.7% [57.6%; 83.7%]

Test of heterogeneity:

Q d.f. p-value

60.87 16 < 0.0001

Details on meta-analytical method:

- Inverse variance method

- DerSimonian-Laird estimator for tau^2

- Trim-and-fill method to adjust for funnel plot asymmetry

> funnel(tf1)


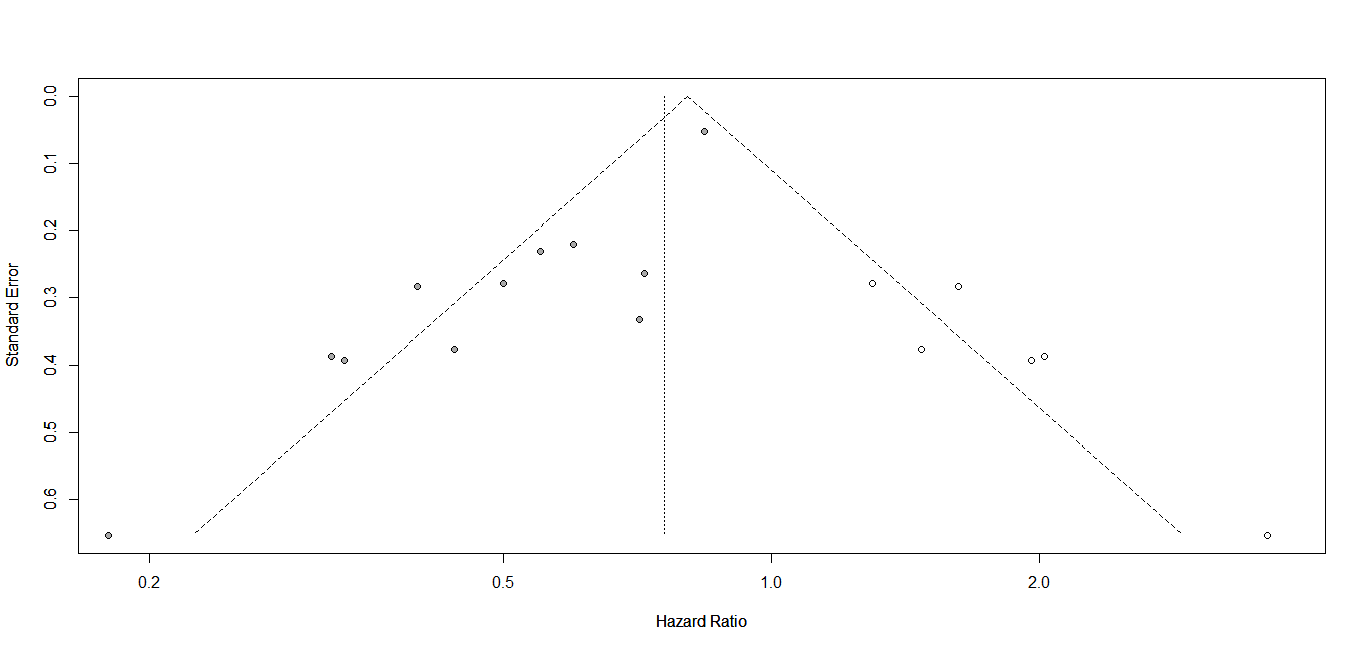

Supplement: S2 File — (DOC) [file pone.0168408.s002.doc]
