# Supplementary material for: Tat-Interacting Protein 30 (TIP30) Expression Serves as a New Biomarker for Tumor Prognosis: A Systematic Review and Meta-Analysis
Source: PLoS One. 2016 Dec 30;11(12):e0168408. doi: 10.1371/journal.pone.0168408 (PMC5201241; doi:10.1371/journal.pone.0168408)
Supplement: S4 File — (DOC) [file pone.0168408.s004.doc]

**S4 file. Quality scale for biological prognostic factors**

(Quoted from “Steels E, Paesmans M, Berghmans T, Branle F, Lemaitre F, Mascaux C, et al. Role of p53 as a prognostic factor for survival in lung cancer: a systematic review of the literature with a meta-analysis. European Respiratory Journal. 2001;18(4):705-719.”)

Except when speciﬁed, the attributed value per item is 2 points if it is clearly deﬁned in the article, 1 point if its description is incomplete or unclear and 0 point if it is not deﬁned or is inadequate.

**Scientiﬁc design**

1. Study objective deﬁnition.

2. Study design: prospective (2 points); retro-spective or retrolective (1 point); not deﬁned (0 point).

3. Outcome deﬁnition.

4. Statistical considerations: fully reported with a preliminary assessment of the patient/sample number to be included and/or analysed (2 points); patient/sample number to be included and/or analysed justiﬁed by the number of studied variables (minimum 10 patients per variable) (1 point); not deﬁned (0 point).

5. Statistical methods and tests description.

**Laboratory methodology**

1. Blinding in the biological assays performance: double-blind (2 points); simple-blind (1 point); unblinded or not deﬁned (0 point).

2. Tested factor description: DNA (types of exons analysed), messenger RNA (complete or partial with description of the primers used), protein (nuclear, cytoplasmic or extracted from cellular components), antibodies (type of tissue or liquid sampled).

3. Tissue sample conservation: either fresh tissue or conservation requiring freezing at ≤-80°C in presence of an anti-RNAase for RNA or freezing at ≤-20°C for DNA, protein and serum, or ﬁxation in formol, alcohol or parafﬁn.

4. Description of the revelation test procedure of the biological factor: PCR with mention of primers, polymerase type, general reaction conditions (concentration of the various reagents, cycle number, duration and temperature of the various steps); DNA-sequencing with the method used, the electrophoresis characteristics (gel composition, duration, temperature and generator voltage) and revelation procedure; reverse transcription (RT) with the transcriptase-reverse type and general conditions of incubation (reagent concentration, temperature and duration); SSCP with the gel composition (acrylamide percentage, glycerol content), other electrophoresis characteristics (duration, temperature and generator voltage), coloration method (the SSCP must be followed by the abnormally migrated fragments sequencing if there is no negative internal control); DGGE with gel composition and gel composition gradient, other electrophoresis characteristics (duration, temperature and generator voltage) and coloration method (the DGGE must be followed by the abnormally migrated fragments sequencing if there is no negative internal control); restricted fragment length polymorphism (RFLP) with the restriction enzyme type, temperature and incubation time, electrophoresis characteristics (gel composition, duration, temperature and generator voltage), coloration method (the RFLP must be followed by the abnormally migrated fragments sequencing if there is no negative internal control); IHC with the ﬁrst antibody type and clone identiﬁcation, second antibody type, reaction characteristics (antibodies concentration, duration and temperature of incubation), colouration method (peroxydase, alkaline phosphatase or chromogenic method), epitope unmasking method in case of ﬁxed tissue, endogenous peroxidase activity inhibition method if the colouration method requires peroxydase; ELISA with the type of antibodies/antigens used, general reaction conditions (reagents concentration, temperature, duration), unspeciﬁc sites blockage (type of serum used), colouration and reading methods (ﬂuorometry, spectrophotometry, etc.); Western blot with types of antibodies/antigens used, gel composition, other electrophoresis characteristics (duration, temperature, generator voltage), colouration method; immunoblot with the type of antibodies/antigens used, the type of membrane, other electrophoresis characteristics (duration, temperature, generator voltage), colouration method.

5. Description of the negative and positive control procedures.

6. Test reproducibility control: between investigators centres if the study is multicentric, or inside the centre if it is unicentric.

7. Deﬁnition of the level of positivity of the test (only evaluated in case of IHC): validated (2 points); arbitrary (1 point); not described (0 point).

**Generalizability**

1. Patient selection criteria, including histological type, disease stage and treatment.

2. Patients’ characteristics, including histology type, disease stage and treatment.

3. Initial workup.

4. Treatment description.

5. Source of samples.

6. Number of unassessable samples with exclusion causes.

**Results analysis**

1. Follow-up description, including the number of events.

2. Survival analysis according to the biological marker.

3. Univariate analysis of the prognostic factors for survival: report of the relative risk with the conﬁdence interval (2 points); results without evaluation of the relative risk and its conﬁdence interval (1 point); not reported or inadequate (0 point).

4. Multivariate analysis of the prognostic factors for survival: report of the relative risk with the conﬁdence interval (2 points); results without evaluation of the relative risk and its conﬁdence interval (1 point); not reported or inadequate (0 point).
